# TRIM8 regulates stemness in glioblastoma through PIAS3‐STAT3

**DOI:** 10.1002/1878-0261.12034

**Published:** 2017-02-15

**Authors:** Changming Zhang, Subhas Mukherjee, Carol Tucker‐Burden, James L. Ross, Monica J. Chau, Jun Kong, Daniel J. Brat

**Affiliations:** ^1^ Department of Pathology and Laboratory Medicine Winship Cancer Institute Emory University School of Medicine Atlanta GA USA; ^2^ Department of Neurosurgery Xiangya Hospital Central South University (CSU) Changsha Hunan China; ^3^ Graduate Program in Cancer Biology Laney Graduate School Emory University Atlanta GA USA; ^4^ Department of Biomedical Informatics Emory University School of Medicine Atlanta GA USA

**Keywords:** glioblastoma, PIAS3, STAT3, stem cell, TRIM8

## Abstract

Glioblastoma (GBM) is the most malignant form of primary brain tumor, and GBM stem‐like cells (GSCs) contribute to the rapid growth, therapeutic resistance, and clinical recurrence of these fatal tumors. STAT3 signaling supports the maintenance and proliferation of GSCs, yet regulatory mechanisms are not completely understood. Here, we report that tri‐partite motif‐containing protein 8 (TRIM8) activates STAT3 signaling to maintain stemness and self‐renewing capabilities of GSCs. TRIM8 (also known as ‘glioblastoma‐expressed ring finger protein’) is expressed equally in GBM and normal brain tissues, despite its hemizygous deletion in the large majority of GBMs, and its expression is highly correlated with stem cell markers. Experimental knockdown of TRIM8 reduced GSC self‐renewal and expression of SOX2, NESTIN, and p‐STAT3, and promoted glial differentiation. Overexpression of TRIM8 led to higher expression of p‐STAT3, c‐MYC, SOX2, NESTIN, and CD133, and enhanced GSC self‐renewal. We found that TRIM8 activates STAT3 by suppressing the expression of PIAS3, an inhibitor of STAT3, most likely through E3‐mediated ubiquitination and proteasomal degradation. Interestingly, we also found that STAT3 activation upregulates TRIM8, providing a mechanism for normalized TRIM8 expression in the setting of hemizygous gene deletion. These data demonstrate that bidirectional TRIM8‐STAT3 signaling regulates stemness in GSC.

**Abbreviations**
GBM: glioblastoma
PIAS3: protein inhibitor of activated STAT3
TRIM8: tri‐partite motif‐containing protein 8

## 1 Introduction

Glioblastoma (GBM) is the most malignant primary brain tumor, with a median survival of 12–15 months following standard therapy that includes resection, chemotherapy, and radiotherapy (Furnari *et al*., 2007; Stupp *et al*., 2005; Wen and Kesari, 2008). GBMs are highly heterogeneous and have diverse tumor microenvironments, numerous forms of neoplastic and host‐derived native and immune cellular constituents, clonal genomic expansions, and a variety of intratumoral gene expression patterns (Wen and Kesari, 2008). Among this widespread heterogeneity is a small subpopulation of glioblastoma stem cells (GSCs) that sits atop the glioma cell hierarchy and regulates global tumor properties of diversity, growth, differentiation, and repopulation following therapy (Dirks, 2010; Reya *et al*., 2001). GSCs are defined by their genetic alterations, cancer‐initiating capabilities, and ability to undergo both self‐renewing division and multilineage differentiation (Park and Rich, 2009; Rosen and Jordan, 2009; Vescovi *et al*., 2006). GSCs can also promote tumor angiogenesis, immune evasion, and resistance to conventional therapies (Bao *et al*., 2006; Calabrese *et al*., 2007; Chen *et al*., 2012). Therefore, identifying regulators that maintain or promote the stem‐like phenotype of GSCs could provide insights for novel therapeutic approaches.

The signal transducer and activator of transcription (STAT) family consists of numerous intracellular proteins sharing the common functional property of signal transmission from growth factor and cytokine receptors through transcriptional regulation; they are often perturbed in cancer in a manner that stimulates growth (Bromberg, 2002; Levy and Darnell, 2002; Yu *et al*., 2014). In GBM for example, STAT3 has been well documented as a facilitator of tumor cell proliferation, invasion, and angiogenesis (Chan *et al*., 2004; Darnell, 2002; Dasgupta *et al*., 2009; Wang *et al*., 2009). More recently, STAT3 has also been demonstrated as an essential factor that maintains the stem cell phenotype of GSCs, in part through its regulation of SOX2, Olig2, and NANOG (Guryanova *et al*., 2011). Others have shown that targeting STAT3 or upstream activators results in disruption of GSC maintenance (Carro *et al*., 2010; Guryanova *et al*., 2011; Herrmann *et al*., 2014). One potent upstream modulator is protein inhibitor of activated STAT3 (PIAS3), which inhibits STAT3 DNA binding and attenuates STAT3‐mediated gene activation (Chung *et al*., 1997). It has been shown that loss of PIAS3 contributes to STAT3 activation and subsequent cell proliferation in many types of cancer, such as malignant mesothelioma, lung carcinoma, and GBM (Brantley *et al*., 2008; Dabir *et al*., 2009, 2014), suggesting that the PIAS3‐STAT3 pathway might also be a signaling pathway that regulates GSC stemness. Potential regulators of the PIAS3‐STAT3 pathway include E3 ubiquitin ligases (Okumura *et al*., 2010; Rodel *et al*., 2000; Saydmohammed *et al*., 2010; Wu *et al*., 2013).

Among the TRIM family, the *TRIM8* gene is aberrantly expressed in high‐grade gliomas and maps to chromosome 10q24.3, a region showing frequent deletion or loss of heterozygosity in GBM (Vincent *et al*., 2000). Interestingly, hemizygous *TRIM8* deletion does not lead to diminished expression, but rather is thought to promote gliomagenesis, leading to the gene product's alternative name, glioblastoma‐expressed RING finger protein (GERP) (Vincent *et al*., 2000). Recently, tri‐partite motif‐containing protein 8 (TRIM8) has been shown to interact with PIAS3, either by causing its degradation through the ubiquitin‐proteasome pathway or through its exclusion from the nucleus, resulting in enhanced STAT3‐dependent signaling in a subset of cancer cell lines (Okumura *et al*., 2010). TRIM8 has also been reported to modulate the transcription of NANOG through its interaction with heat shock 90β in embryonic stem cells, suggesting a regulatory role in stem cell self‐renewal (Okumura *et al*., 2011). Based on the functional importance of STAT3 in GSCs, its known regulation by PIAS3, and the unexplored role of TRIM8 in regulating stem cell properties in GBM, we investigated the regulation of STAT3‐mediated support of stem cell properties in GSC by TRIM8.

## 2 Materials and methods

### 2.1 Investigations using TCGA GBM data

We downloaded the copy number alterations (CNA) for the *TRIM8* gene and the mRNA expression data for the genes, *NESTIN*,* SOX2*,* STAT3*,* Olig2*,* NANOG*,* BMI*, and *TRIM8* by U133 microarray using the Glioblastoma TCGA Provisional dataset accessed from the cBioPortal for Cancer Genomics website (http://www.cbioportal.org/ on September 8, 2015). We tested for the Pearson and Spearman correlations for the gene pairs of *TRIM8* and the stem cell markers listed above. The resulting Pearson and Spearman correlation coefficients are reported as *r* = correlation coefficient; *P* =*P*‐value; and 95% confidence interval (lower, upper).

### 2.2 Isolation and culture of GBM‐derived neurosphere cells

Normal human neural progenitor cells (NHNP) were obtained from Lonza (Basel, Switzerland) (Martinez *et al*., 2006; PT‐2599), and GBM‐derived neurosphere cells were isolated and characterized from GBM patient samples and designated as N08‐30, N08‐74, N09‐32, N12‐115, N12‐159, and N13‐213, as described previously (Chen *et al*., 2014; Mukherjee *et al*., 2016). Both the N12‐159 and N13‐213 harbor chromosome 10q (*PTEN*) loss, but have no *EGFR* amplification. N08‐30 has both EGFR amplification and 10q (*PTEN*) loss. The diagnosis of GBM was established by senior neuropathologist (DJB) in accordance with WHO criteria. The stem cell phenotype of neurospheres was confirmed by stem cell marker expression, the neurosphere self‐renewal assay, and degree of differentiation. Mycoplasma was tested using Lonza Mycoplasma Detection Kit (LT07‐703). GBM‐derived neurosphere cells and NHNP cells were cultured in Neurobasal‐A media (Invitrogen, Waltham, MA, USA) with the growth factors, FGF, EGF, and heparin as described previously (Mukherjee *et al*., 2016).

### 2.3 Reverse transcription PCR

Total cellular RNA was isolated with an RNeasy kit (QIAGEN#74134; QIAGEN, Venlo, Netherlands) and reverse‐transcribed into cDNA using the First‐strand Synthesis Kit (Invitrogen#12328‐040). Reverse transcription PCR was performed on an Eppendorf Mastercycler using Platinum PCR Supermix (Invitrogen#12532‐016) with the following primers: TRIM8: forward 5′‐CCTATCTGCCTGCACGTTTT‐3′ and reverse 5′‐GTTGTAGGCCTGGTTGCACT‐3′; GFP: forward 5′‐CAGCCACAACGTGTACATCC‐3′ and reverse 5′‐GGTCCTTGCTGATCTTGGTC‐3′; TRIM8‐GFP: forward 5′‐CCGCAAGATTCTCGTCTGTT‐3′ and reverse 5′‐CATGGCGCTCTTGAAGAAGT‐3′; GAPDH: forward 5′‐ACTTTGGTATCGTGGAAGGA‐3′ and reverse 5′‐TTGACAAAGTGGTCGTTGAG‐3′.

### 2.4 Immunocytochemistry

GBM cell lines were cultured to 90% confluence and dispersed into single cells using Accutase. Cells were resuspended in 4% paraformaldehyde in PBS for fixation. Fixed cells were spun down onto slides using cytospin and were washed with 0.3% Triton X‐100 in PBS (PBST). Cells were blocked with 0.1% BSA in PBST and incubated with primary antibody against SOX2 and TRIM8 at 4 **°**C overnight. Cells were washed in PBST and incubated with secondary antibody for two hours at room temperature. After washing with PBST, cells were mounted with hardset Vectashield mounting media with DAPI.

### 2.5 Immunohistochemical staining of tissue samples

Paraffin‐embedded tissues were placed in a 60 °C oven for 1 h. Deparaffinization was performed with xylene, 100%, 95%, 70%, and 50% alcohol and washed in tap water. Antigen retrieval was performed using TRS diluted 1 : 10. The tissues were placed in solution and heated to 120 °C using Biocare Decloaking Chamber (Biocare, Concord, CA, USA). Tissues were cooled, gently washed in tap water, and placed in TBS IHC wash buffer. Endogenase peroxidase was blocked with 3% H_2_O_2_. Tissues were incubated with antibodies diluted with diluent at room temperature (DAKO, Santa Clara, CA, USA). HRP antibody was used for visualization and developed with DAB. Counterstain was performed with hematoxylin for 3–5 min. Tissues were dehydrated, cleared in xylene, mounted, and coverslipped.

### 2.6 Lentiviral particle generation and infection

*TRIM8* cDNA was obtained from *pLenti‐TRIM8‐mGFP* TrueORF clone (Origene#RC205812L2; Origene, Rockville, MD, USA), with the vector control *mGFP* cDNA from *pLenti‐C‐mGFP* TrueORF clone (Origene#PS100071). The TRIM8 shRNA lentiviral plasmid in *pGFP‐C‐shLenti* vector was obtained from Origene (Martinez *et al*., 2006; TL300821). Lentivirus packaging plasmids *psPAX2* (Addgene#12260; Addgene, Cambridge, MA, USA) and *pMD2.G* (Addgene#12259) were cotransfected with cDNA or shRNA into HEK293T cells with Lipofectamine 3000 transfection reagents (Martinez *et al*., 2006; L3000015). Supernatant was collected and concentrated by precipitation with PEG‐it virus precipitation solution (Cat#LV810A‐1; SBI, Palo Alto, CA, USA) according to the protocol. Viral pellets were resuspended in 1× PBS and kept at −80 °C for stock or directly used in cell transduction. Gene expression efficiency was confirmed by GFP expression under fluorescence microscopy and western blot analysis.

### 2.7 Flow cytometry and FACS

To obtain GBM stem cells, we flow‐sorted patient‐derived neurosphere cells for CD133‐positive cells on the BD FACS Canto II (BD Biosciences, San Jose, CA, USA). To analyze stem cell marker expression in TRIM8‐manipulated neurosphere cells, we performed flow cytometry in BD FACS Canto II (BD Biosciences). Antibodies used included SOX2‐APC‐conjugated (Miltenyi Biotec, Bergisch Gladbach, Germany) and CD133‐PE‐conjugated (Miltenyi Biotech). TRIM8 expression was assessed using primary antibody anti‐TRIM8 (NBP1‐89776; NOVUS, Littleton, CO, USA) and goat anti‐rabbit PE‐conjugated, goat anti‐rabbit APC‐conjugated secondary antibody (Thermo scientific, Waltham, MA, USA), respectively.

### 2.8 Western blot analysis

Western blots were performed using 10% standard gels from Bio‐Rad (Hercules, CA, USA). Proteins were isolated from cells as described (Mukherjee *et al*., 2016). The following antibodies were applied for western blot analysis or IF analysis: TRIM8 (NBP1‐89776; NOVUS), PIAS3 (9042; Cell Signaling, Danvers, MA, USA), STAT3 and phosphor‐STAT3 (PSTAT3‐Y705) (Cell Signaling), GFP (TA180076; Origene), β‐actin (A5441; Sigma, St. Louis, MO, USA), GAPDH (GTX627408; GeneTex, Irvine, CA, USA), GFAP (3670; Cell Signaling), NESTIN (ab6320; Abcam, Eugene, OR, US), SOX2 (2748; Cell Signaling), CD133 (W6B3C1; Miltenyi), and c‐MYC (ab32072, Abcam).

### 2.9 Differentiation assay

GBM‐derived neurosphere cells were cultured on Poly‐D‐lysine‐coated culture dishes or slides and induced for differentiation via withdrawal of bFGF and EGF growth factor by adding 4% serum in DMEM. At specific time points, cells were harvested for ICC staining or western blot analysis as described above (Guryanova *et al*., 2011).

### 2.10 Neurosphere formation assay

GBM neurospheres were dispersed by Accutase (AT‐104) into single cells, and 10^4^ cells were seeded in triplicate into six‐well plates in Neurobasal culture medium. At indicated time points, neurosphere formation was measured by infinity analyze software (Lumenera Corporation, Ottawa, ON, Canada) under Olympus microscope (Tokyo, Japan).

### 2.11 Extreme limiting dilution assay (ELDA)

We used extreme limiting dilution assay to investigate whether TRIM8 overexpression induces stemness in N08‐30 TRIM8 and N12‐159 TRIM8 overexpression lines along with their respective vector controls. We collected cell pellets and established single‐cell suspension in media using Accutase. Finally, we plated serially diluted cells in 96‐well plates, with a maximum of 100 cells per well and a minimum of one cell per well. We cultured the cells for 10 days at 37 **°**C. For each dilution series, we counted wells that showed sphere formation on day 11. Data were analyzed and displayed using ELDA software available at http://bioinf.wehi.edu.au/software/elda/.

### 2.12 IL‐6 and STAT3 inhibition experiments

Human interleukin 6 (IL‐6) was obtained from Cell Signaling (Cat#8904) and reconstituted in 0.1% BSA solution as 100 μg·mL^−1^ concentration stock. GBM neurospheres were treated with 10 ng·mL^−1^ or 50 ng·mL^−1^ of IL‐6 for 48 h, and proteins were collected for western blot analysis. Selective STAT3 Inhibitor XI was obtained from Calbiochem (San Diego, CA, USA) (Martinez *et al*., 2006; STX‐0119) and reconstituted in DMSO as 10 mm concentration stock solution. GBM neurosphere cells were treated with either 10 μm or 100 μm for 24 h, and proteins were collected for western blot analysis.

### 2.13 Statistical analysis

flowjo (Ashland, OR, USA) and graphpad (La Jolla, CA, USA) were used for statistical analysis. All grouped data are presented as mean ± standard deviation (SD). Results were analyzed for significance by one‐way analysis of variance (Guryanova *et al*., 2011) or nonparametric *t*‐test using GraphPad Prism.

## 3 Results

### 3.1 TRIM8 is expressed in GBMs despite hemizygous deletion and expression levels correlate with stem cell markers

**Figure 1:**
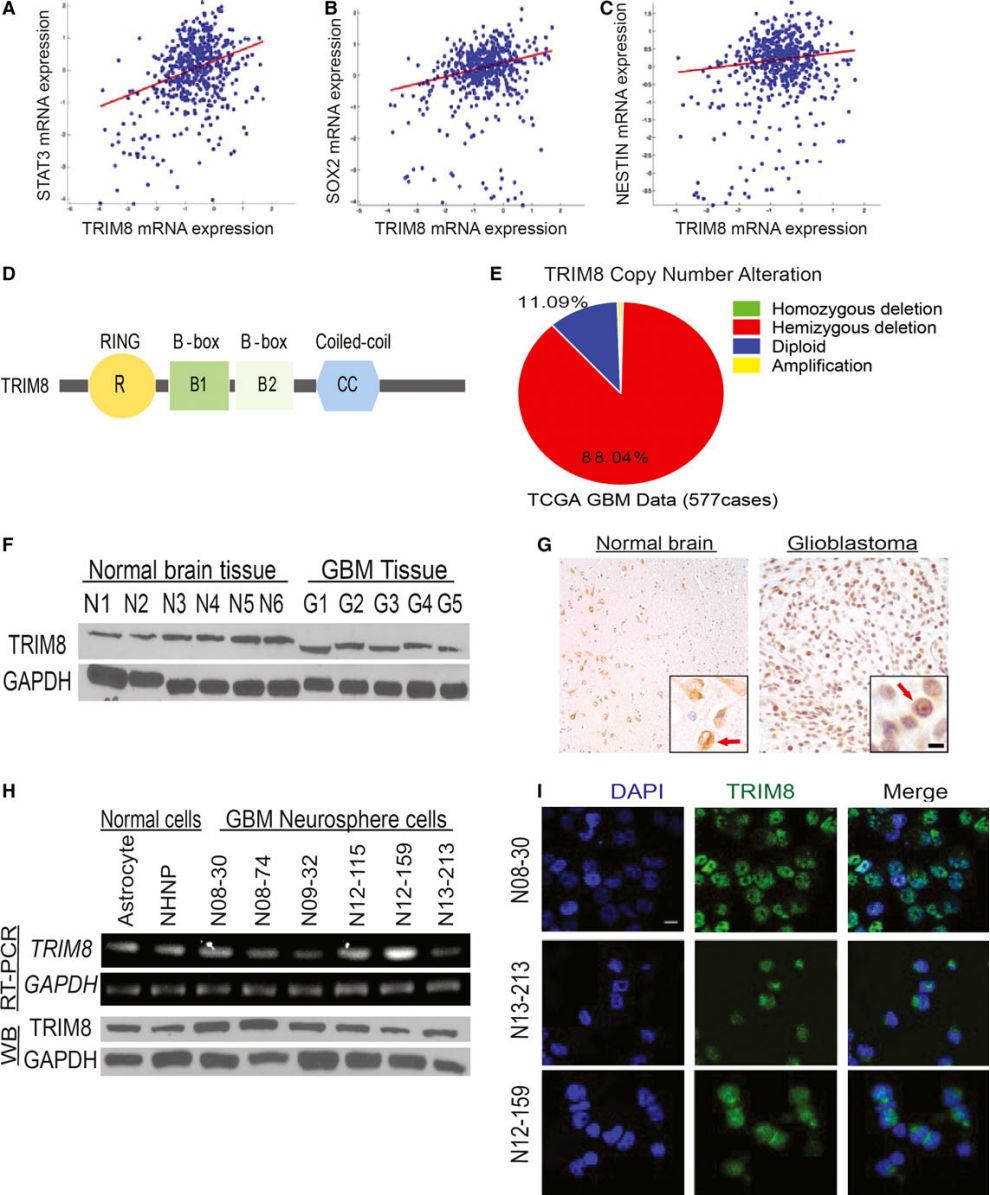
TRIM8 is expressed in GBM samples and neurosphere cells. (A–C) Gene expression correlation of *TRIM8* with *STAT3*,*SOX2*, and *NESTIN* using U133 mRNA data from TCGA. *TRIM8* vs *STAT3*: Pearson's correlation coefficient: *r* = 0.36, *P* < 0.00001. *TRIM8* vs *SOX2*: Pearson's correlation coefficient: *r* = 0.22, *P *< 0.00001. *TRIM8* vs *NESTIN*: Pearson's correlation coefficient: *r *= 0.22, *P *< 0.00001. (D) Schematic representation of TRIM8 protein with RING, B‐box, and coiled‐coil domains. (E) TRIM8 copy number variation among 577 GBM samples in TCGA dataset. TRIM8 shows hemizygous deletion in 88.04% of cases. (F) Western blot analysis of TRIM8 protein among six normal human brain tissues (N1–N6) and five GBMs (G1–G5). (G) Immunohistochemical (IHC) staining of TRIM8 in normal brain and GBM tissues. TRIM8 (+) cells (indicated by arrow) are predominantly neurons in the normal brain and show cytoplasmic staining. TRIM8 expression in GBM is predominant in nuclei of tumor cells with modest cytoplasmic staining. Sections were counterstained with hematoxylin. Scale bar = 10 μm. (H) Reverse transcription PCR analysis and western blot analysis of TRIM8 among two normal cell lines and six patient‐derived GBM neurosphere cells. NHNP = normal human neural progenitor cell. (I) Immunocytochemical (ICC) staining of TRIM8 in neurosphere cells showing nuclear expression of TRIM8. TRIM8 was labeled in GFP, and nuclei were counterstained with DAPI (blue). Scale bar = 10 μm.

We were interested in identifying genes that were consistently associated with stem cell signatures in GBM and could potentially have regulatory properties and identified TRIM8. To assess whether TRIM8 expression correlates with global measures of GBM stemness in human samples, we investigated the gene expression of *TRIM8* and other transcription factors or stem cell markers, including *STAT3*,* SOX2*, and *NESTIN*, using data from the TCGA GBM database. *TRIM8* expression was strongly and positively correlated with *STAT3*,* SOX2*,* NESTIN* (Fig. 1A–C), as well as *Olig2*,* Nanog*, and *BMI* (not shown), suggesting that TRIM8 might be mechanistically relevant to GBM stemness. TRIM8 was first identified as GERP (Vincent *et al*., 2000) and contains one RING finger domain, which has ubiquitin ligase (E3) function, two B‐box domains, one coiled‐coil domain, and a C‐terminal domain (Fig. 1D). Further investigation of *TRIM8* using CNA data within the TCGA revealed that 88% of GBMs showed hemizygous deletion of *TRIM8* (Fig. 1E), consistent with previous reports and the location of *TRIM8* on chromosome 10q24.3, which is frequently deleted in *IDH* wild‐type GBMs (Vincent *et al*., 2000). We next checked TRIM8 protein expression in normal brain samples and GBM tissues and found no significant difference between normal brain and GBM tissue (Fig. 1F), despite chromosome 10 deletion in all GBM tissue samples (Table S2). Similarly, TRIM8 was identified by immunohistochemical staining of patient‐derived GBM samples (Fig. 1G). Whereas the normal brain showed modest cytoplasmic expression of TRIM8 in neurons, GBMs showed predominantly nuclear expression in neoplastic cells, with less cytoplasmic expression. To further confirm that TRIM8 is equally expressed in normal and GBM tissues, we investigated TRIM8 expression in two non‐neoplastic cell lines (human astrocytes and normal human neural progenitors; NHNPs) and six patient‐derived GBM neurosphere lines. Although chromosome 10 was deleted in five GBM neurosphere lines (Table S1), TRIM8 showed similar mRNA and protein expression among the non‐neoplastic and neoplastic glial cell lines (Fig. 1H). Immunocytochemical staining of neurosphere cells showed that TRIM8 was expressed most strongly in the nuclei, with fainter cytoplasmic staining (Fig. 1I), also consistent with previous findings (Reymond *et al*., 2001). Taken together, we found that TRIM8 has similar expression levels in GBM samples as in normal brain, despite its frequent hemizygous deletion in GBM, which might suggest a necessary function, and our correlative data from the TCGA raise the possibility of GSC regulation.

### 3.2 TRIM8 promotes stemness and self‐renewal capacity of GBM‐derived neurospheres

**Figure 2:**
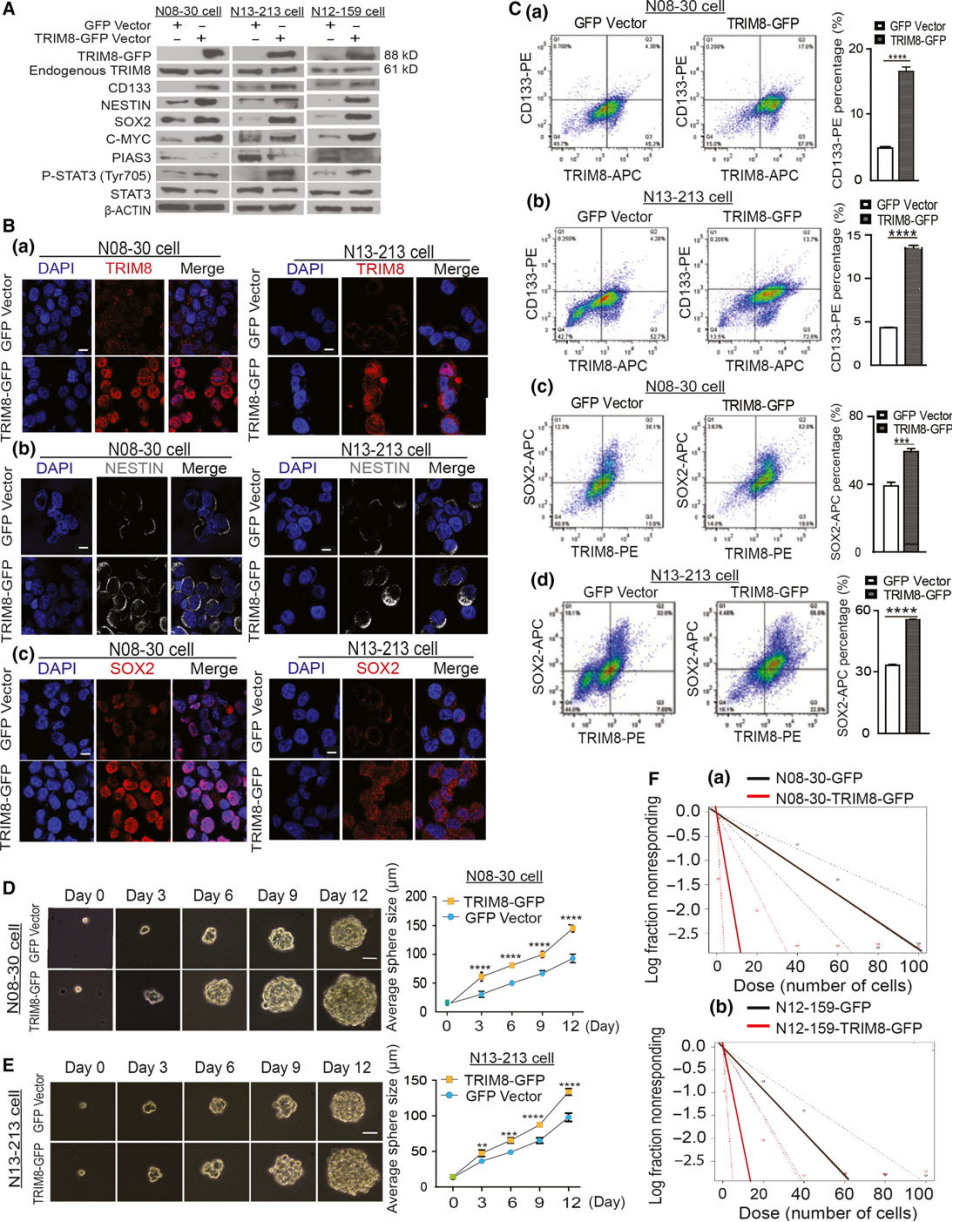
Ectopic expression of TRIM8 enhances stemness and self‐renewal of GBM‐derived neurospheres. (A) Western blot analysis shows upregulation of CD133, NESTIN, SOX2, and c‐MYC following ectopic expression of TRIM8‐GFP fusion protein. WB also shows reduced PIAS3 and increased phosphorylated STAT3 (Tyr705) and total STAT3 following TRIM8 overexpression. (B) ICC staining of TRIM8 (red) with stem cell markers NESTIN (white), and SOX2 (red) in neurosphere cells overexpressing TRIM8. Nuclei were counterstained with DAPI (blue). Scale bar = 10 μm. (C) Flow cytometric analysis and statistical analysis showing CD133 and SOX2 upregulation in neurosphere cells with TRIM8 overexpression. CD133 was tagged by PE (red: 555 nm) with TRIM8 tagged by APC (blue: 676 nm). SOX2 was tagged by APC (blue: 676 nm) with TRIM8 tagged by PE (red: 555 nm). ***P < 0.001, ****P < 0.0001. Statistics: Data are means ± SD (*n* = 3), by nonparametric *t‐*test. (D,E) Effects of TRIM8 overexpression on neurosphere formation. Representative images of neurospheres expressing GFP vector or TRIM8‐GFP vector at specific points. Scale bar = 30 μm. Statistical analysis of average sphere size: by one‐way analysis of variance (ANOVA). Data are means ± standard deviation (SD) (*n* = 6). **P < 0.01, ***P < 0.001, ****P < 0.0001. (F) Extreme limiting dilution assay (ELDA) shows that TRIM8 overexpression reduces the cell dose required for colony formation, supporting enhanced stem cell properties.

To determine whether TRIM8 affects measures of stemness in GSCs, we created stable patient‐derived GBM neurosphere cells that express TRIM8 protein tagged with GFP and compared them to those transfected with GFP alone. RT‐PCR demonstrated that transfected *TRIM8‐GFP* and *GFP* were expressed in N08‐30 cells (Fig. S1A). Western blots confirmed that the TRIM8‐GFP fusion protein was present in addition to the endogenous TRIM8 (Fig. 2A). Using this model system with upregulated TRIM8 expression in three GBM neurosphere cell lines, we found that TRIM8 expression was associated with upregulation of the GSC markers CD133 and NESTIN, as well as the stem cell transcription factors SOX2 and c‐MYC (Fig. 2A). We also performed immunocytochemical staining of N08‐30 cells and N13‐213 cells that expressed either GFP or TRIM8‐GFP and found that c‐MYC (Fig. S1B), NESTIN, and SOX2 (Fig. 2B: b,c) were elevated in those cells with stable ectopic overexpression of TRIM8 (Fig. 2B: a). Using flow cytometric analysis, we also found that those GBM neurospheres that overexpress TRIM8‐GFP (Fig. S1C: a–d) showed upregulation of the GSC marker CD133 (Fig. 2C: a–b) and SOX2 (Fig. 2C: c,d).

We next examined whether TRIM8 overexpression influenced stem cell‐related functions of GBMs. One standard measure of assessing proliferation and self‐renewal capacity of GSCs *in vitro* is the neurosphere formation assay (Guryanova *et al*., 2011). Patient‐derived GBM cells are plated in a dispersed manner and followed for their ability to form spheres. We found that cells overexpressing TRIM8 consistently developed into spheres more quickly and also developed larger neurospheres (Fig. 2D,E). We extended these studies by performing an extreme limiting dilution assay (ELDA) to determine whether TRIM8 overexpression promotes self‐renewal properties in neurosphere lines. Cells with greater stem‐like properties require fewer numbers to establish colonies within a given time period. We found that TRIM8 overexpression significantly reduced the cell dose required for colony formation, indicating an increase in stem cell properties (Fig. 2F: a,b). We also directly measured the proliferation marker Ki67 by western blot and demonstrated that it was elevated in cells that overexpressed TRIM8 (Fig. S1D) compared to controls. Collectively, these data demonstrate that TRIM8 expression is associated with the expression of stem cell markers and transcription factors in GBM neurospheres and enhances stem cell functions.

### 3.3 Knockdown of TRIM8 impairs GBM neurosphere stemness and self‐renewal capacity

**Figure 3:**
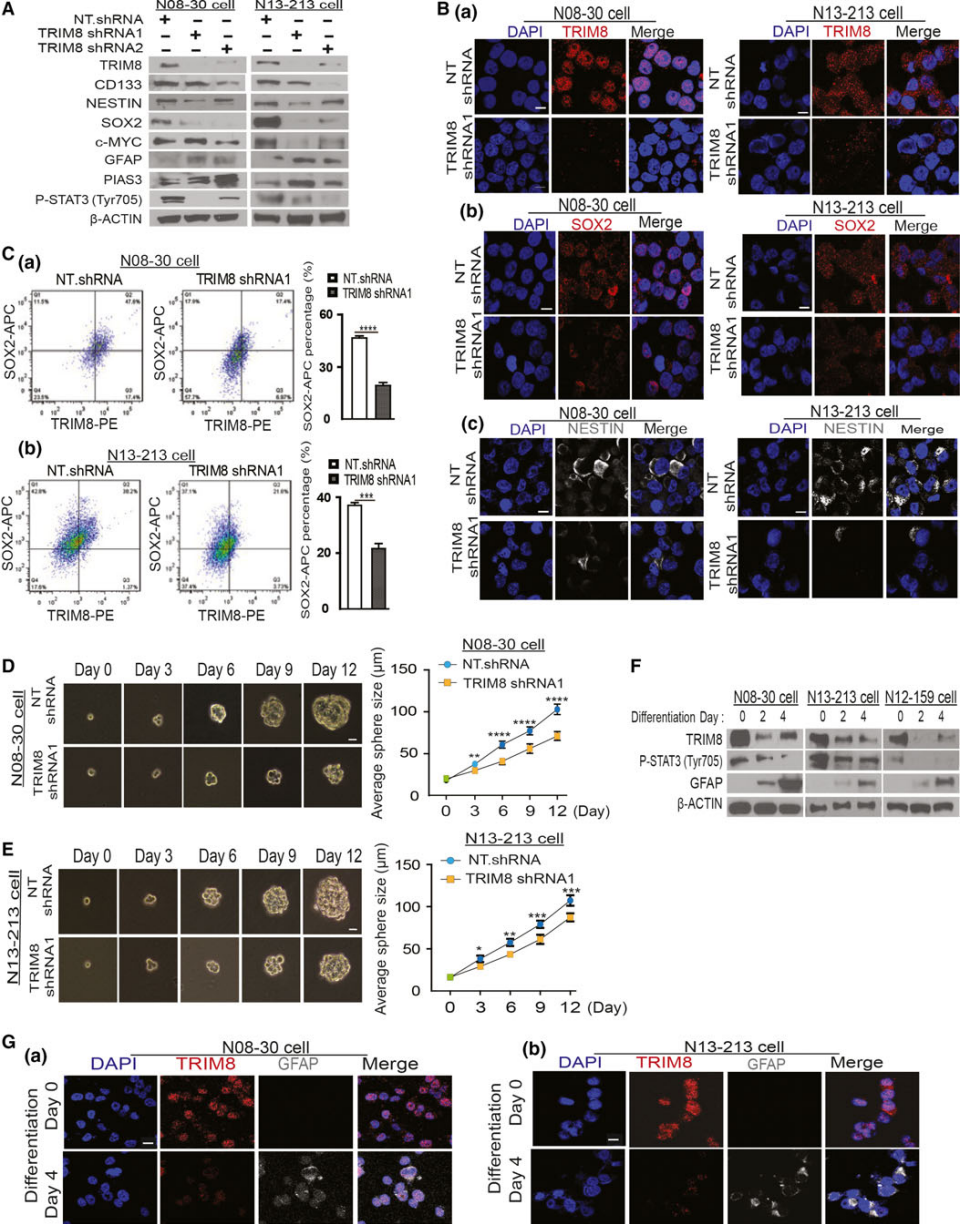
Knockdown of TRIM8 promotes differentiation and impairs GBM stemness and self‐renewal capacity. (A) Western blot analysis shows reduced CD133, NESTIN, SOX2, and c‐MYC, and upregulated GFAP in GBM neurosphere cells following TRIM8 knockdown by TRIM8 shRNA1 or TRIM8 shRNA2. NT shRNA = nontargeting control shRNA. Western blot also shows upregulated PIAS3 and reduced phosphorylated STAT3 (Tyr705) following shRNA knockdown of TRIM8. (B) ICC shows reduced expression of TRIM8 (red), NESTIN (white), and SOX2 (red) in TRIM8 shRNA1‐knockdown cells compared to NT shRNA cells. Nuclei were counterstained with DAPI (blue). Scale bar = 10 μm. (C) Flow cytometric analysis and statistical analysis of SOX2 expression in TRIM8‐overexpressing neurosphere cells. SOX2 was tagged by APC (blue: 676 nm) with TRIM8 tagged by PE (red: 555 nm). ***P < 0.001, ****P < 0.0001. Statistics: Data are means ± SD (*n* = 3), by nonparametric *t*‐test. (D,E) TRIM8 knockdown impairs neurosphere formation. Representative images of neurosphere expressing NT shRNA and TRIM8 shRNA1 at specific time points are shown. Scale bar = 30 μm. Statistical analysis of average sphere size: by one‐way analysis of variance (ANOVA). Data are means ± standard deviation (SD) (*n* = 6). **P < 0.01, ***P < 0.001, ****P < 0.0001. (F) Western blot analysis shows reduced TRIM8 and activated STAT3 (Tyr705), with increased GFAP expression following differentiation induced by serum. (G) ICC reveals reduced TRIM8 (red) and increased GFAP (white) after four days of differentiation induced by serum. Nuclei were counterstained with DAPI (blue). Scale bar = 10 μm.

Because TRIM8 overexpression was associated with stem cell features in GBM neurospheres, we next interrogated effects of TRIM8 knockdown on the maintenance of GBM neurosphere stemness. Here, one nontargeted (NT) shRNA and two short hairpin RNAs (shRNA) specifically targeted to *TRIM8* were transduced into neurosphere cell lines to study effects of TRIM8 downregulation. RT‐PCR and western blots demonstrated that transfection of TRIM8 shRNA was effective in downregulating TRIM8 mRNA and protein expression in GBM neurosphere lines (Figs 3A and S2A). We also found that knockdown of TRIM8 reduced NESTIN and SOX2 expression while partially and variably downregulating CD133 and c‐MYC expression (Fig. 3A). Immunocytochemical staining also revealed lower expression of NESTIN, SOX2 (Fig. 3B), and c‐MYC (Fig. S2B) after targeting cells with shRNA for TRIM8. By flow cytometry, we found that cells transfected with shRNA directed at TRIM8 showed lower expression of TRIM8 (Fig. S2C,E) and also showed reduced levels of the stem cell markers SOX2 and NESTIN (Figs 3C: a,b and S2D,F), with the percentage of TRIM8‐APC decreased from 64.2% to 27.5% (Fig. S2C) in flow cytometric analysis. Similar results were found using the N13‐213 cell line (Fig. 3C: b and S2E).

To gain a better understanding of the effects of TRIM8 knockdown on neurosphere self‐renewal capacity, we examined neurosphere formation and found that TRIM8 shRNA1‐expressing cells grew much slower than control NT shRNA cells at all time points in two different cell lines (Fig. 3D,E). The expression of proliferation marker Ki67 was also reduced, indicating lower cell division following TRIM8 knockdown (Fig. S2G). To further elucidate the relationship between TRIM8 and GSC stemness and differentiation, we examined the effects of serum‐induced differentiation on TRIM8 expression. Following 48 h of culture in serum‐containing media to induce differentiation, we found that the expression of GFAP was increased in all three cell lines tested and confirmed the findings by immunocytochemical staining (Figs 3G and S2H). Under these same conditions, we noted that the level of activated STAT3 [pSTAT(Tyr705)] was reduced (Fig. 3F). Interestingly, we also found that GFAP was increased after TRIM8 knockdown (Fig. 3A), indicating that the downregulation of TRIM8 correlates with the loss of GSC stemness and the enhancement of GSC differentiation. Taken together, knockdown of TRIM8 in GBM neurosphere cells impairs GSC stemness and promotes differentiation.

### 3.4 TRIM8 regulates GBM neurosphere stemness through PIAS3 and STAT3

**Figure 4:**
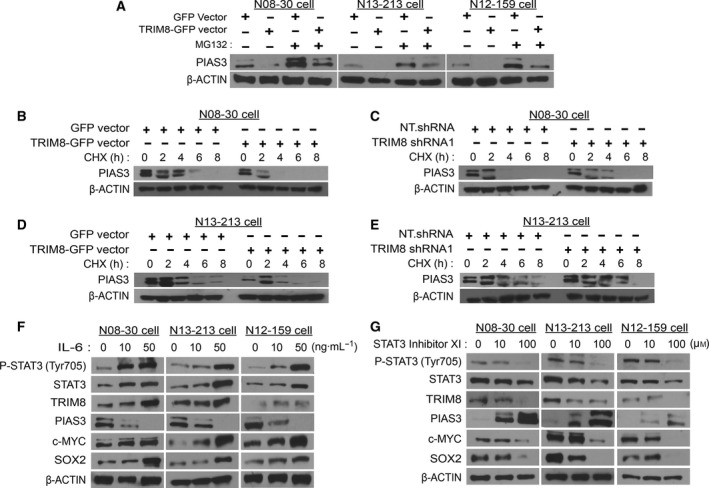
TRIM8 modulates GBM stemness via a positive loop of PIAS3‐STAT3 pathway. (A) Western blot analysis of PIAS3 accumulation following TRIM8 overexpression in the presence of MG132 (20 μm) for four hours. (B–E) Western blot analyses showing that the half‐life of PIAS3 was reduced by TRIM8 overexpression and increased by TRIM8 knockdown after treating cells with 100 μg·mL^−1^ cycloheximide (CHX) for 0, 2, 4, 6, and 8 h in two different neurosphere cell lines. (F) Western blot showing that interleukin 6 (IL‐6; 10 and 50 ng·mL^−1^) induced phosphorylated STAT3 (Tyr705) and also enhanced expression of STAT3, c‐MYC, SOX2, and TRIM8. (G) Western blot showing that the STAT3 Inhibitor XI (STX‐0119; 10 and 100 μm) selectively suppresses phosphorylated STAT3 (Tyr705) and reduces expression of STAT3, c‐MYC, SOX2, and TRIM8.

Previous studies have reported that TRIM8 negatively regulates PIAS3 in cancer cell lines (Okumura *et al*., 2010). It is also known that loss of PIAS3 in GBM contributes to STAT3 activation and subsequent cell proliferation (Brantley *et al*., 2008). Based on this, we hypothesized that the intermediary PIAS3 is suppressed by TRIM8 to upregulate STAT3. To test this, we used patient‐derived GBM neurospheres that overexpress TRIM8‐GFP and found that PIAS3 expression is suppressed compared to GFP‐expressing controls. We also showed that STAT3 is upregulated and activated under these same conditions in three different GBM neurosphere cell lines (Fig. 2A). Conversely, after knockdown of TRIM8, PIAS3 expression was increased and the expression levels of activated STAT3 were reduced (Fig. 3A). Because it has been reported that TRIM8 directs the ubiquitination through its RING finger domain and leads to the ubiquitin‐mediated proteasomal degradation of PIAS3 (Okumura *et al*., 2010), we tested the effects of the ubiquitin‐proteasome pathway inhibitor MG132 on the expression of PIAS3. In the presence of MG132, we found that PIAS3 protein levels are increased compared to nontreated controls. We also showed that TRIM8 overexpression is associated with suppression of MG132‐induced increases in PIAS3 (Fig. 4A), indicating that TRIM8 overexpression antagonizes the effects of the inhibitor, likely due to enhanced E3‐ubiquitin‐mediated degradation of PIAS3. To further investigate the expression of PIAS3 protein in the presence of TRIM8, we studied the protein stability following inhibition of protein synthesis by cycloheximide (CHX). In the presence of CHX, we found that the half‐life of PIAS3 was approximately 4 h (Fig. 4B,D). When TRIM8 was overexpressed, the half‐life of PIAS3 was reduced to 2 h, suggesting that TRIM8 enhances the degradation of PIAS3. We used shRNA to knock down TRIM8, and we found the reverse: suppression of TRIM8 significantly prolonged the half‐life of PAIS3 in the presence of CHX (Fig. 4C,E). Taken together, our data indicate that TRIM8 suppresses PIAS3, most likely through ubiquitin‐mediated proteasomal degradation, leading to upregulation and activation of STAT3.

To explain how TRIM8 protein expression is maintained in GBMs that have hemizygous deletion of its gene (Fig. 1E–H), we considered that there might be a positive feedback loop between STAT3 and TRIM8 that could contribute, because the strong positive mRNA correlation between *TRIM8* and *STAT3* could be due to bidirectional regulation. Our investigation of the *TRIM8* promoter for potential transcriptional regulators used patch1.0 software (GeneXplain GmbH, Wolfenbüttel, Germany) based on binding sites from TRANSFAC^®^ Public 6.0 library on the BIOBOSE website. We found two potential transcription factor binding sites (TFBS) for STAT3 in the *TRIM8* promoter region with binding sequences of TTCCCGGAA (http://www.gene-regulation.com/pub/databases.html). The presence of STAT3 TFBS in TRIM8 promoter was also confirmed independently by QIAGEN (http://www.sabiosciences.com/chipqpcrsearch.php?species_id=0&factor=STAT3&gene=TRIM8&nfactor=n&ninfo=n&ngene=n&B2=Search) and based on SABioscience's proprietary database. Similarly, we identified multiple c‐MYC and OCT1 TFBS in the TRIM8 promoter region using patch1.0 software, and confirmed with QIAGEN. Thus, STAT3 could directly activate TRIM8 transcription or accomplish this through c‐MYC and OCT1.

**Figure 5:**
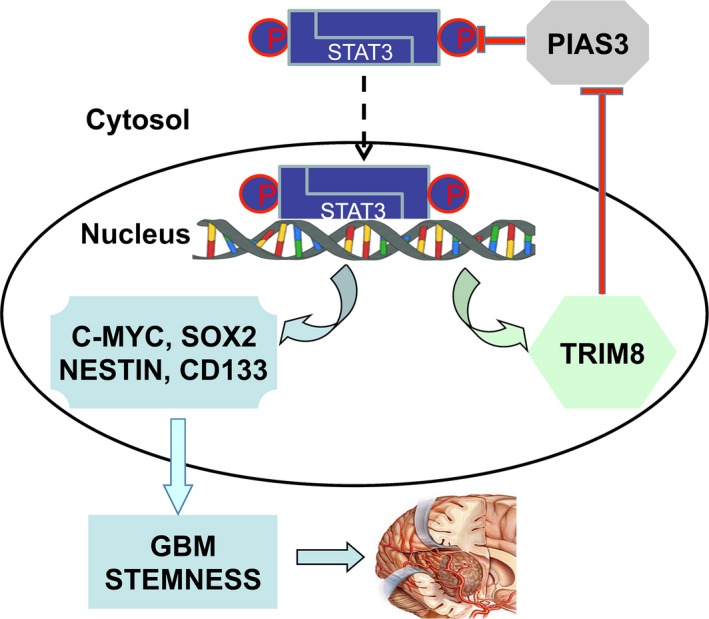
Schematic model of the TRIM8‐PIAS3‐STAT3 pathway that regulates glioblastoma stemness. TRIM8 suppresses PIAS3 through proteasomal degradation, resulting in enhanced activation of STAT3. STAT3 promotes GSC stemness factors SOX2, NESTIN, CD133, and c‐MYC. In addition, STAT3 promotes TRIM8 expression, forming a positive bidirectional regulatory loop involving TRIM8 and STAT3.

It is known that interleukin 6 (IL‐6) potently induces STAT3 activation in GSC and regulates their self‐renewal capacity through IL‐Rα binding (Penuelas *et al*., 2009; Wang *et al*., 2009). Therefore, we examined the impact of IL‐6‐induced activation of STAT3 on TRIM8 expression. We treated cells with increasing concentrations of IL‐6 and found that STAT3 phosphorylation increased in a dose‐dependent manner. We also found that the stem cell markers SOX2 and c‐MYC were upregulated in a similar dose‐dependent manner. Interestingly, TRIM8 showed a parallel dose‐dependent increase in protein expression in response to IL‐6 (Fig. 4F), suggesting that STAT3 activation contributes to TRIM8 expression in GBM neurosphere cells. As the potential target of TRIM8, PIAS3 showed reduced expression that correlated with TRIM8 upregulation (Fig. 4F). A small‐molecule STAT3 Inhibitor XI (STX‐0119) has been reported to selectively suppress STAT3 and its target genes both *in vitro* and *in* *vivo* (Ashizawa *et al*., 2011; Matsuno *et al*., 2010). After treating cells with STX‐0119, we found that phosphorylated STAT3, SOX2, and c‐MYC were suppressed. Interestingly, TRIM8 was also suppressed and PIAS3 expression increased (Fig. 4G), indicating that selective targeting of STAT3 activation with STX‐0119 can suppress TRIM8 expression and other stem cell markers in GBM neurosphere cells (Guryanova *et al*., 2011). Collectively, our data demonstrate that TRIM8 regulates GBM neurosphere stemness through a bidirectional positive loop involving PIAS3 and STAT3 (Fig. 5).

## 4 Discussion

Our investigation, as well as those of others, has shown that *TRIM8* is frequently deleted in GBM (Vincent *et al*., 2000), yet despite this, does not show downregulation at the transcriptional or translational level, but rather shows variable expression and activity that supports the stem cell phenotype of GSC. The finding of gene deletion and maintained protein expression is not novel, because other single copy gene losses have been demonstrated in the setting of constant protein expression, yet it raises interesting questions related to transcriptional regulation following gene deletion (Geiger *et al*., 2010). In the TCGA GBM data, TRIM8 expression was highly correlated with nearly all stem cell markers and transcription factors that we investigated, leading to our hypothesis that TRIM8 may have a functional role in supporting stem cell properties in this tumor type. As TRIM8 has E3 ubiquitin ligase activity, similar to other RING‐containing TRIM family members, we focused on potential targets that might negatively regulate STAT3, a potent regulator of GSC that was highly correlated with TRIM8 in TCGA data.

In patient‐derived GBM neurosphere cells, we showed that TRIM8 expression is inversely correlated with the expression of PIAS3, a potent inhibitor of STAT3, and is likely due to ubiquitination of PIAS3 by the RING finger domain of TRIM8, which possesses E3‐ubiquitin ligase activity. Aside from TRIM8, PIAS3 is not known to be a target of ubiquitination by other ubiquitin ligases, although this has not been fully evaluated. However, TRIM8 has been shown to target other proteins for proteasomal degradation, including TAK1 as a component of NF‐κB regulation (Li *et al*., 2011). By way of suppressing PIAS3, we also demonstrated that TRIM8 leads to upregulation and activation of STAT3 and promotes expression of numerous GSC‐related transcription factors, including c‐MYC and SOX2, leading to the enhanced self‐renewal capacity and upregulation of stem cell markers CD133 and NESTIN (Fig. 5). Moreover, we also demonstrated that knockdown of TRIM8 impairs GBM stemness and self‐renewal ability and leads to glial differentiation.

Mechanisms that activate TRIM8 or cause its increased expression in GSCs are not established. IFN‐γ is capable of increasing gene expression of TRIM8 mRNA (Toniato *et al*., 2002) and could accomplish this by activating the JAK‐STAT pathway or independently. Interestingly, we also showed that STAT3 activation induced by IL‐6 leads to enhanced TRIM8 expression and that suppressing TRIM8 expression attenuates STAT3 activation. In our search for potential transcriptional factor binding sites (TFB) for STAT3, we found two in the *TRIM8* promoter region. Similarly, we identified multiple c‐MYC and OCT1 TFBs. Thus, STAT3 could directly activate TRIM8 transcription or accomplish this through c‐MYC and OCT1. This unexpected positive feedback between STAT3 and TRIM8 may explain the phenomenon of TRIM8 protein expression level despite hemizygous *TRIM8* gene deletion in GBM. Mechanisms leading to transcriptional upregulation following gene deletion need further exploration.

Only recently have investigations focused on the TRIM family of proteins as potential regulators of tumor development and progression (Hatakeyama, 2011). Increasing evidence now indicates that TRIM proteins also play an important role in stem cell biology. For example, TRIM28 functions as a transcriptional corepressor in orchestrating the primer binding site‐mediated silencing of integrated M‐MLV proviruses in mouse embryonic carcinoma and embryonic stem cells (Wolf and Goff, 2007), while TRIM19 (PML) regulates asymmetric division of hematopoietic stem cells via PPAR‐δ‐FAQ pathway (Ito *et al*., 2012). Our laboratory has demonstrated that TRIM3 regulates asymmetric cell division in normal neural progenitor cells as well as in GSCs (Chen *et al*., 2014) and maintains stem cell equilibrium by regulating active NOTCH1 nuclear transportation (Mukherjee *et al*., 2016). In addition to the functions demonstrated in our current study, TRIM8 also regulates NANOG through its translocation of STAT3 in mouse embryonic stem cells (Okumura *et al*., 2011).

Our findings support the role of TRIM8 as a potential oncogene in GBM through its regulation of PIAS3‐STAT3 and GSC stemness, rather than a tumor suppressor, as might be expected by its frequent deletion. Previous studies supporting TRIM8 as an oncogene have shown that its expression enhances cell growth *in vitro* through PIAS3‐STAT3 signaling in HepG2 cells, HeLa cells, and NIH3T3 cells (Okumura *et al*., 2010) and regulates TNF‐induced NF‐κB pathway as an oncogene in MCF7 and HeLa cells (Tomar *et al*., 2012). In contrast, TRIM8 inhibits renal cell carcinoma formation *in vitro* (Caratozzolo *et al*., 2014), suggesting that TRIM8 might also act as a tumor suppressor in some settings and may be context dependent in its function. A recent publication suggested that TRIM8 might act as a tumor suppressor in GBM (Micale *et al*., 2015), although these studies did not confirm the protein expression of TRIM8. Our data clearly established that the hemizygous deletion of *TRIM8* is not associated with reduced protein expression. Additionally, we explored a novel relationship between TRIM8 and stemness using CD133‐sorted GSC cultures as our model, which has the advantage of a highly enriched stem cell population (eightfold enriched CD133 expression) as compared to studies in U87MG, which are not optimal for studying stem cell properties (Annabi *et al*., 2009).

STAT3 is one of the best studied oncogenic signaling nodes, and it exerts enormous influence on tumor growth properties, including the support of stemness in diverse cancer types including those of breast, prostate, and brain (Dasgupta *et al*., 2009; Marotta *et al*., 2011; Schroeder *et al*., 2014; Wang *et al*., 2009). Because targeting STAT3 or its crucial upstream activators results in disruption of GSC maintenance, many have focused on this pathway as a potential therapeutic target (Carro *et al*., 2010; Guryanova *et al*., 2011; Herrmann *et al*., 2014). Our finding of the TRIM8‐STAT3 positive feedback loop in GBM provides new insight into the regulation of STAT3 and offers other opportunities for the development of treatments that impair stemness in GSC and potentially in other cancers that show TRIM8 overactivity.

Based on our findings, many biological and clinical implications arise. First, our finding established that targeting TRIM8 reduces GSC stem cell marker expression and self‐renewal capacity, suggesting that TRIM8 and STAT3 signaling pathways are potential therapeutic targets for GBM. Second, our findings add TRIM8 to a growing list of upstream pathways that promote STAT3 activation in GSCs, including IL‐6, Notch, PI3K, BMX, EZH2, and a number of receptor tyrosine kinases (Bromberg *et al*., 1999; Fan *et al*., 2010; Guryanova *et al*., 2011; Kim *et al*., 2013). Targeting either TRIM8 or STAT3 could effectively disrupt the positive feedback loop and attenuate the self‐renewal capacity and tumor propagation of GBM. In conclusion, we demonstrated that TRIM8 contributes to maintenance of stemness and self‐renewal capacity in GSC through its activation of STAT3.

## Author Contributions

CZ, SM, and DJB involved in conception and design and in analysis and interpretation of data. CZ, SM, CTB, JLR, MJC, JK, and DJB involved in data acquisition and in writing and reviewing of the manuscript. CZ, SM, and CTB involved in development of methodology. CTB and DJB provided administrative, technical, or material support. CZ and DJB supervised the study.

## Supporting information


**Fig. S1.** (A) Reverse transcriptional PCR analysis of TRIM8‐GFP, GFP, intrinsic TRIM8 and GAPDH in N08‐30 cells. (B) ICC staining of c‐MYC (red) in TRIM8 overexpression neurosphere cells. Nuclei were counterstained with DAPI (blue). Scale bar = 10 μm. (C) Statistical analysis of TRIM8 expression in TRIM8 overexpressing neurosphere cells quantitated from flow cytometry. TRIM8 was tagged by APC (blue: 676 nm) or by PE (red: 555 nm). **P < 0.01, ***P < 0.001, ****P < 0.0001. Statistics: Data are means ± SD (*n* = 3), by nonparametric *t*‐test. (D) Western blot analysis of Ki67 in TRIM8 overexpressing neurosphere cells showing increased expression in cell expressing TRIM8‐GFP.
**Fig. S2.** (A) Reverse transcriptional PCR analysis of TRIM8 in N08‐30 cells that included untreated controls, NT shRNA (nontargeted) and TRIM8 shRNA. (B) ICC showing reduced c‐MYC (red) in GBM neurosphere cells following TRIM8 knockdown. Nuclei were counterstained with DAPI (blue). Scale bar = 10 μm. (C, E) Statistical analysis shows reduced TRIM8 expression by flow cytometry in TRIM8 knockdown neurosphere cells. TRIM8 was tagged by PE (red: 555 nm). ***P < 0.001, ****P < 0.0001. (D, F) Flow cytometry and statistical analysis of NESTIN expression in TRIM8 shRNA treated neurosphere cells. NESTIN was tagged by APC (blue: 676 nm) with TRIM8 tagged by PE (red: 555 nm). ***P < 0.001, ****P < 0.0001. Statistics: Data are means ± SD (*n* = 3), by nonparametric *t*‐test. (G) Western blot analysis of Ki67 (proliferation maker) following TRIM8 knockdown in GBM neurosphere cells. (H) ICC showing reduced TRIM8 (red) expression and increased GFAP (white) in GBM neurosphere cells at days 0 and 4 following differentiation induced by serum. Nuclei were counterstained with DAPI (blue). Scale bar = 10 μm.
**Table S1.** Chromosomal copy number alterations of GBM neurosphere cell lines.
**Table S2.** Chromosomal copy number alterations of GBM tumor samples.
