# Supplementary material for: TRIM8 regulates stemness in glioblastoma through PIAS3‐STAT3
Source: Mol Oncol. 2017 Feb 15;11(3):280–94. doi: 10.1002/1878-0261.12034 (PMC5332279; doi:10.1002/1878-0261.12034)
Supplement: Supplementary file 1 — Fig. S1. (A) Reverse transcriptional PCR analysis of TRIM8‐GFP, GFP, intrinsic TRIM8 and GAPDH in N08‐30 cells. (B) ICC staining of c‐MYC (red) in TRIM8 overexpression neurosphere cells. Nuclei were counterstained with DAPI (blue). Scale bar = 10 μm. (C) Statistical analysis of TRIM8 expression in TRIM8 overexpressing neurosphere cells quantitated from flow cytometry. TRIM8 was tagged by APC (blue: 676 nm) or by PE (red: 555 nm). **P < 0.01, ***P < 0.001, ****P < 0.0001. Statistics: Data are means ± SD (n = 3), by nonparametric t‐test. (D) Western blot analysis of Ki67 in TRIM8 overexpressing neurosphere cells showing increased expression in cell expressing TRIM8‐GFP. Fig. S2. (A) Reverse transcriptional PCR analysis of TRIM8 in N08‐30 cells that included untreated controls, NT shRNA (nontargeted) and TRIM8 shRNA. (B) ICC showing reduced c‐MYC (red) in GBM neurosphere cells following TRIM8 knockdown. Nuclei were counterstained with DAPI (blue). Scale bar = 10 μm. (C, E) Statistical analysis shows reduced TRIM8 expression by flow cytometry in TRIM8 knockdown neurosphere cells. TRIM8 was tagged by PE (red: 555 nm). ***P < 0.001, ****P < 0.0001. (D, F) Flow cytometry and statistical analysis of NESTIN expression in TRIM8 shRNA treated neurosphere cells. NESTIN was tagged by APC (blue: 676 nm) with TRIM8 tagged by PE (red: 555 nm). ***P < 0.001, ****P < 0.0001. Statistics: Data are means ± SD (n = 3), by nonparametric t‐test. (G) Western blot analysis of Ki67 (proliferation maker) following TRIM8 knockdown in GBM neurosphere cells. (H) ICC showing reduced TRIM8 (red) expression and increased GFAP (white) in GBM neurosphere cells at days 0 and 4 following differentiation induced by serum. Nuclei were counterstained with DAPI (blue). Scale bar = 10 μm. Table S1. Chromosomal copy number alterations of GBM neurosphere cell lines. Table S2. Chromosomal copy number alterations of GBM tumor samples. [file MOL2-11-280-s001.docx]

**Supplemental Data:**

**Figure legends:**

**Figure S1:** (A) Reverse transcriptional PCR analysis of TRIM8-GFP, GFP, intrinsic TRIM8 and GAPDH in N08-30 cells. (B) ICC staining of c-MYC (red) in TRIM8 overexpression neurosphere cells. Nuclei were counterstained with DAPI (blue). Scale bar = 10μm. (C) Statistical analysis of TRIM8 expression in TRIM8 overexpressing neurosphere cells quantitated from flow cytometry. TRIM8 was tagged by APC (blue: 676nm) or by PE (red: 555nm). **P<0.01, ***P<0.001, ****P<0.0001. Statistics: Data are means$\pm$SD (n=3), by non-parametric t-test. (D) Western blot analysis of Ki67 in TRIM8 overexpressing neurosphere cells showing increased expression in cell expressing TRIM8-GFP.

**Figure S2:** (A) Reverse transcriptional PCR analysis of TRIM8 in N08-30 cells that included untreated controls, NT-shRNA (non-targeted) and TRIM8 shRNA. (B) ICC showing reduced c-MYC (red) in GBM neurosphere cells following TRIM8 knockdown. Nuclei were counterstained with DAPI (blue). Scale bar = 10μm. (C, E) Statistical analysis shows reduced TRIM8 expression by flow cytometry in TRIM8 knockdown neurosphere cells. TRIM8 was tagged by PE (red: 555nm). ***P<0.001, ****P<0.0001. (D, F) Flow cytometry and statistical analysis of NESTIN expression in TRIM8 shRNA treated neurosphere cells. NESTIN was tagged by APC (blue: 676nm) with TRIM8 tagged by PE (red: 555nm). ***P<0.001, ****P<0.0001. Statistics: Data are means$\pm$SD (n=3), by non-parametric t-test. (G) Western blot analysis of Ki67 (proliferation maker) following TRIM8 knockdown in GBM neurosphere cells. (H) ICC showing reduced TRIM8 (red) expression and increased GFAP (white) in GBM neurosphere cells at days 0 and 4 following differentiation induced by serum. Nuclei were counterstained with DAPI (blue). Scale bar = 10μm.

**Supplemental Figure S1**

**
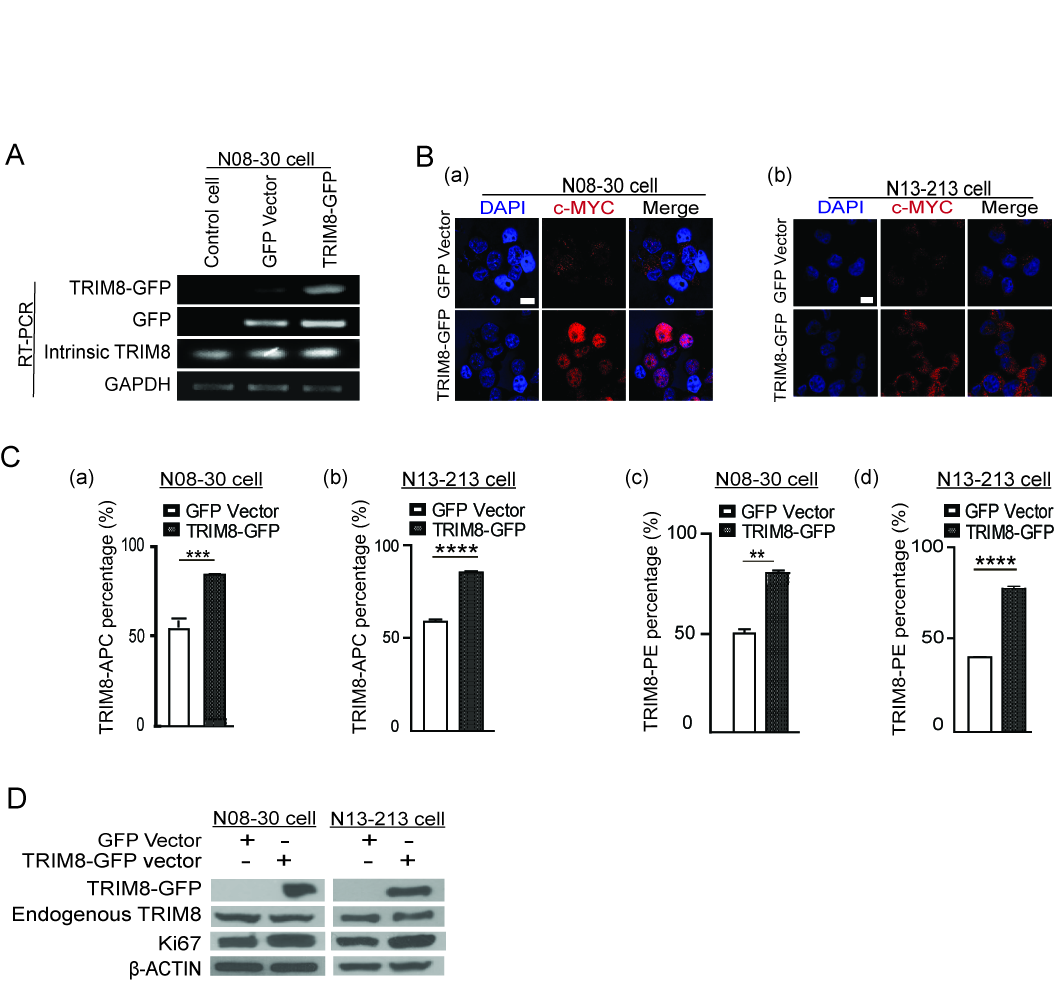
**

**Supplemental Figure S2**

**
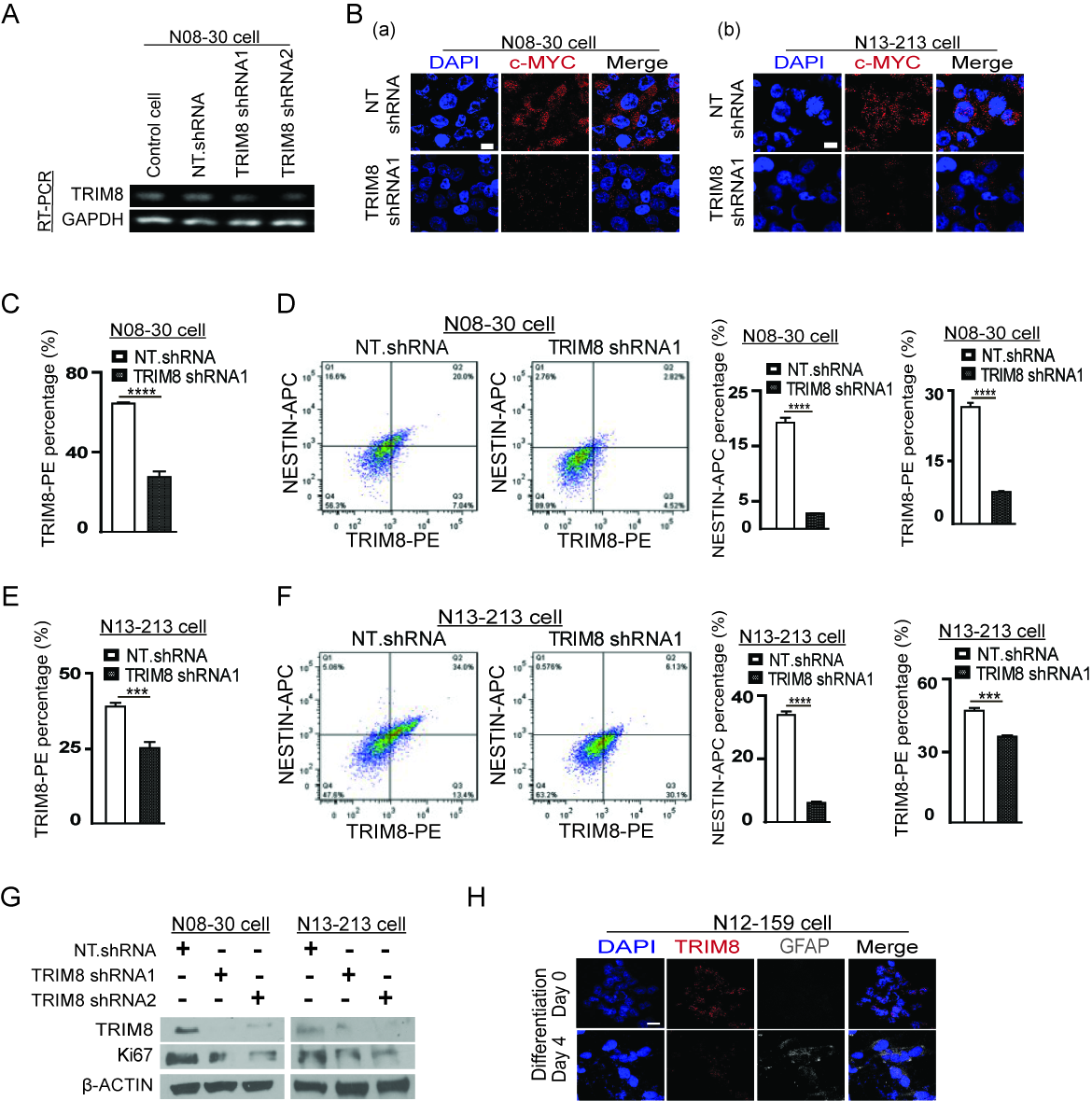
**

**Supplemental Table legends:**

**Supplemental Table 1: Chromosomal Copy Number Alterations of GBM Neurosphere Cell Lines**

Table 1 shows 6 neurosphere lines containing Copy Number Alteration status. All neurosphere lines except N08-74 show Chromosome 10 deletion. All neurosphere lines except N09-32 show chromosome 7 gain, but only N08-30 contains EGFR amplification.

**Supplemental Table 2: Chromosomal Copy Number Alterations of GBM Tumor Samples**

Table 2 shows the Copy Number Alteration data of 5 GBM samples. All samples have chromosome 10 deletion.

**Supplemental Table 1: Chromosomal Copy Number Alterations of GBM Neurosphere Cell Lines**

**Supplemental Table 2: Chromosomal Copy Number Alterations of GBM Tumor samples**

| **Neurosphere ID** | **Chrom. 10** | **Chrom. 7** | **EGFR WT vs. Amp** |
| --- | --- | --- | --- |
| N08-30 | Deleted | Gain | Amplified |
| N08-74 | NA | Gain | WT |
| N09-32 | Diploid | Diploid | WT |
| N12-115 | Deleted | Gain | WT |
| N12-159 | Deleted | Gain | WT |
| N13-213 | Deleted | Gain | WT |

| **GBM Tumor sample #** | **Chrom. 10** | **Chrom. 7** | **EGFR WT vs. Amp** |
| --- | --- | --- | --- |
| G1 | Deleted | Diploid | WT |
| G2 | Deleted | Gain | Amplified |
| G3 | Deleted | Diploid | WT |
| G4 | Deleted | Gain | Amplified |
| G5 | Deleted | Gain | Amplified |
